# Tribological Properties of Al-Based Composites Reinforced with Fullerene Soot

**DOI:** 10.3390/ma14216438

**Published:** 2021-10-27

**Authors:** Firuz Yunusov, Tatiana V. Larionova, Oleg Tolochko, Alexander D. Breki

**Affiliations:** Institute of Machinery, Materials and Transport, Peter the Great St. Petersburg Polytechnic University, 195251 Saint-Petersburg, Russia; larionova@hotmail.com (T.V.L.); ol_tol@hotmail.com (O.T.); albreki@yandex.ru (A.D.B.)

**Keywords:** composite, powder metallurgy, tribological properties, scanning electron microscopy

## Abstract

Aluminum-based composite materials reinforced with fullerene soot, which is a mixture of fullerene and amorphous carbon, are promising materials for friction and wear applications. Composite materials: aluminum with 2% fullerene soot (f.s) and Al with 2% f.s and 2% Cu were obtained via mechanical milling followed by hot pressing. The tribological properties (friction and wear) of the listed composites were investigated and compared with the results for pure aluminum obtained under similar conditions. It has been shown that the addition of fullerene soot reduces the friction coefficient by 25%. At additional alloying with copper, the friction coefficient decreased by 35% in comparison with pure aluminum and also lad to a decrease in friction fluctuations. The wear rate of composite Al with 2% f.s decreased twice in comparison with that of pure aluminum, and with additional alloying it decreased 2.5 times. The morphology of the wear surfaces was investigated by scanning electron microscopy (SEM). The influence of fullerene soot and additional alloying on the wear mechanism was shown.

## 1. Introduction

Friction and wear have a huge impact on the energy used by and resource conservation of production. A total of 20% of the world’s total energy consumption is used to overcome friction and 3% is used for reconstruction and equipment replacement due to wear [1]. Friction and wear management are increasingly recognized as key energy efficiency strategies not only in macro-scale moving machinery, but also in micro/nanoscale technologies. Through the use of specially developed surface treatment, materials, and lubricant technologies to reduce friction and wear, energy losses can be reduced in the long term by up to 40% [2].

One of the ways to reduce friction and wear is the development of new composite materials with good tribological properties. The main service characteristics of the antifriction materials are: low friction coefficient in conditions of imperfect lubrication, resistance to setting and seizure during friction, high wear resistance, thermal conductivity, resistance to compression and creep, fatigue strength, and resistance to corrosion [3].

Aluminum alloys have many of these properties and are used as antifriction materials. The use of aluminum as a base for antifriction material is limited because of its low hardness and, therefore, low wear resistance and extremely high reactivity. If the oxide film is destroyed during the sliding process, the clean aluminum surface instantly adheres to the surface of the counterbody. If the sliding continues, the material will undergo intense adhesive wear or the rubbing surfaces will become completely jammed. Therefore, aluminum alloy plain bearings are used only in conditions where there is satisfactory lubrication.

Metal-matrix composites containing carbon nanostructures, due to their high hardness and self-lubrication effect, can be considered as a promising solution to provide better wear resistance [4,5,6,7,8,9,10]. It was noted in [4] that in composites, carbon nanotubes prevented the direct contact of two sliding surfaces due to the formation of lubricating film and showed better wear resistance compared to other dispersed particles. The addition of 4% carbon nanotubes leads to a decrease in the friction coefficient by 55%. In [5], it was found that CNT-reinforced composites showed a lower wear rate and friction coefficient compared to pure aluminum under soft wear conditions. However, under hard wear conditions these composites showed a higher coefficient of friction and wear rate compared to pure aluminum. It was also found that the friction and wear of CNT-reinforced composites are highly dependent on the applied load, and there is a critical load above which CNTs can have an adverse effect on the wear resistance of aluminum [5].

The reduction in the friction coefficient and wear rate by 21.7% compared to the basic Al6061 alloy was observed for the Al6061-based composites with the addition of 0.25% graphene due to their self-lubrication function and higher hardness. These results showed that the material has a high resistance to abrasive wear [6].

Although the good mechanical properties of Al-based composites with the addition of fullerenes have been repeatedly reported in the literature [11,12,13,14,15,16], there are few studies on the effect of fullerene soot on the physical and mechanical properties of metal-matrix composites.

The aim of the work is to study the tribological properties of aluminum-based composites reinforced with fullerene soot.

## 2. Materials and Methods

**Figure 1:**
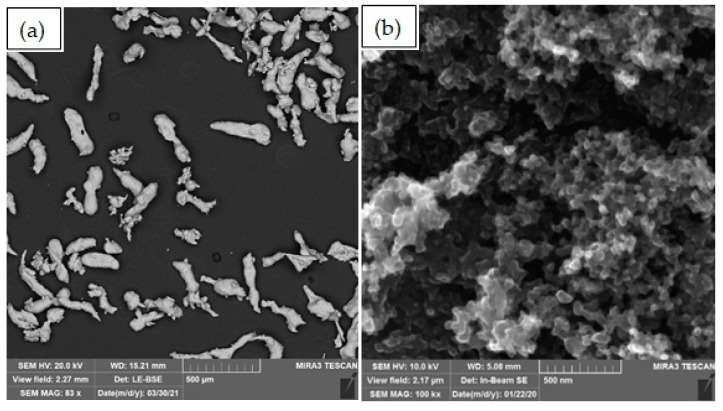
SEM images of pure materials: (**a**) aluminum, (**b**) fullerene soot.

In order to obtain composite materials, composite powder was prepared by mechanical milling. As an initial component, aluminum powder of grade PA-4 GOST 6058-73 with a particle size of up to 125 microns was used. Fullerene soot (f.s.) containing up to 30% fullerene C_60_ was provided by Suzhou Dade Carbon Nanotechnology Co (Suzhou, China). SEM images of the starting materials are shown in Figure 1. Other technological additions are stearic acid (1 wt.%) and a mixture of CaCl_2_, NaCl, and KCl in the ratio 2:1: (3 wt.%).

**Figure 2:**
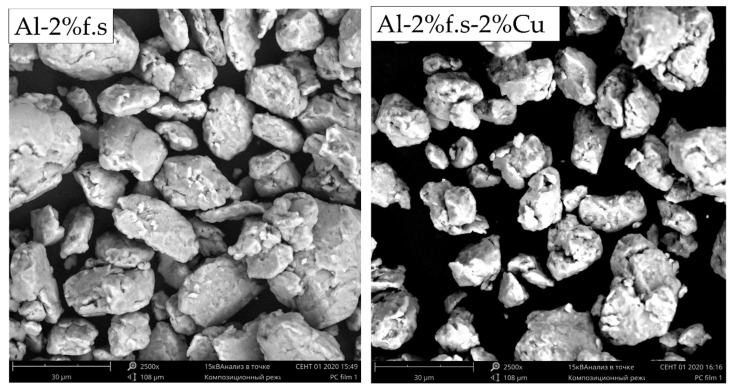
SEM images of the composite powders.

Ball milling was carried out in a Fritsch Pulverisette 7 premium line ball mill (FRITSCH, Markt Einersheim, Germany) in 80 ml steel containers. Initially, the powders were mixed at a speed of 200 rpm for one hour, then milled at 600 rpm for two hours in an inert atmosphere. The ratio of the mass of the grinding balls to the mass of the powder was 10:1. The milling regimes were chosen on the basis of preliminary work. Figure 2 shows SEM images of the composite powders.

Powder compacting was carried out in two stages. In the first stage, the powder was subjected to cold pressing at a pressure of 400 MPa, then after heating the mold to a temperature of 480 °C and holding it there, hot pressing was carried out at a pressure of 200 MPa. The manufactured specimens were cylinders that were 40 mm in diameter and approximately 6 mm in height.

The hydrostatic density was measured according to (1) after the samples had been coated with wax. A GH-202 scale (A&D Company, Tokyo, Japan) with an AD-1653 kit was used

(1) $$\rho_{hydr}=\frac{M_{1}\rho_{w}\rho_{p}}{\left(M_{2}-M_{3}\right)\rho_{p}-M_{4}\rho_{w}}$$

where: *M*_1_ mass of the sample without a protective layer, g; *M*_2_ mass of the sample with a protective layer suspended in air, g; *M*_3_ mass of the sample with protective layer suspended in liquid, g; *M*_4_ mass of the protective layer, g; *ρ*_w_ water density, g/mm^2^; *ρ_p_* density of the protective layer, g/mm^2^. Theoretically estimated densities were calculated using the mixture rule.

The surfaces of the specimens were mechanically ground sequentially with 400, 600, 1200, and 2500 grit papers. Vickers hardness was measured at a load of 100 N and dwelling time of 10 s on a ZWICKZHU 250 (ZwickRoell, Ulm, Germany). The friction test was performed on a DHR-2 rheometer (TA Instruments, New Castle, USA) at room temperature. A “ring-on-plate” friction scheme was used. The counterbody was made of stainless steel of grade 202. The outer diameter of the ring was 31.8 mm and the inner diameter was 28.6 mm. A series of tests carried out at different sliding speeds were performed at a constant load of 20 N (0.13 MPa) at sliding speeds of 0.015, 0.03, 0.06, 0.12, 0.18, 0.24, 0.30, and 0.33 m/s. Before the testing, preliminary friction testing was performed at a speed of 0.15 m/s and load of 20 N for 10 min. At least six experiments per point were performed and the standard deviation was calculated. The friction force dependent on the applied load was measured at 10 N (0.07 MPa), 15 N (0.10 MPa), 20 N (0.13 MPa), and 25 N (0.16 MPa) loads at a sliding speed of 0.12 m/s. The temperature change in the contact area during the test was measured with a chromel−alumel thermocouple (SmartModule, Zvenigovo, Russia) attached to an Aktakom recorder-converter (Eliks, Moscow, Russia).

**Figure 3:**
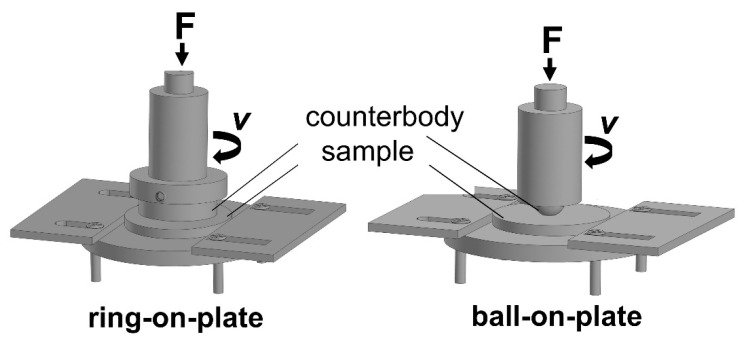
Schemes of the wear test.

A wear resistance test was carried out on a PBD-40 machine (Bosh, Gerlingen, Germany). Two methods were used: the first method measured the mass loss of the specimen when it was tested according to the “ring-on-plate” scheme; the second measured the diameter of the wear spot, when tested, according to the “ball-on-plate” scheme. The schemes of the wear test are shown in Figure 3.

In the “ring-on-plate” test, the counterbody was the same as that used in the friction test (described in Figure 3). The rotation speed was 200 rpm (3.2 m/s) with a load of 96 N (0.65 MPa). The duration of the experiment was 15 min. The worn volume was calculated by mass loss and measured density of the specimens and then normalized by the sliding distance and applied load. When testing according to the “ball-on-plate” scheme, a steel ball of grade ШX 15 (Russian Federatin Standard GOST) with a diameter of 12.7 mm was used as a counterbody. The rotation speed was 200 rpm and a load of 96 N was applied (calculated hertzian contact stress 1161 MPa). Wear volume was calculated as spherical cap volume using the measured diameter of the wear hole. The duration of each experiment was 15 min. It should be mentioned that even the rotation and the applied load are the same for the cases of ring and ball counterbodies, the contact area is completely different.

Scanning electron microscopy (SEM) and energy dispersion spectroscopy (EDS) were performed on a MIRA 3 TESCAN microscope (Tescan, Brno-Kohoutovice, Czech Republic) with an Oxford Instruments X-Max 80 EDS detector. (Oxford Instruments plc., Abingdon, UK)

## 3. Results and Discussion

The experimentally measured densities and hardness of all compact samples are shown in Table 1. Al-initial and Al-milled samples were prepared for reference, the former from initial pure aluminum powder and the latter from preliminary milled pure aluminum powder.

The relative density of all materials was above 98%. This implies that well-compacted samples with acceptable percentages of voids and porosity were obtained.

Table 1 shows the average Vickers microhardness values for all samples. It is noticeable that the hardness of the Al-milled samples was increased by 50% compared to the sample obtained from the initial aluminum powder. This is due to the effect of strain-hardening, as described in [17,18]. The increase in hardness with the addition of fullerene soot and copper is described in detail in our previous work [19].

**Figure 4:**
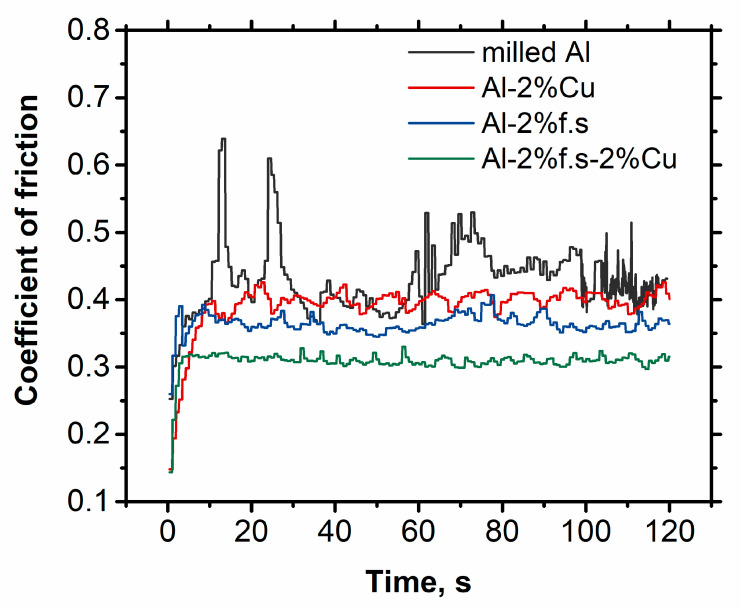
The dependence of the friction coefficient on time at a sliding speed of 0.12 m/s and at a load of 20 N (0.13 MPa).

The dependence of friction on time for the different samples is presented in Figure 4. The highest coefficient of friction was observed for compact-milled aluminum and the lowest for the composite Al-2%f.s-2%Cu. This can be explained by the higher hardness as well as the self-lubricating effect. It is known that the carbon film can cover the wear surface and act as a solid lubricant, reducing friction. The greatest frictional fluctuations were observed in compact pure aluminum, while the Al-2%f.s-2%Cu composite had the smallest fluctuations. This can be explained by its more homogeneous structure and high hardness [19].

**Figure 5:**
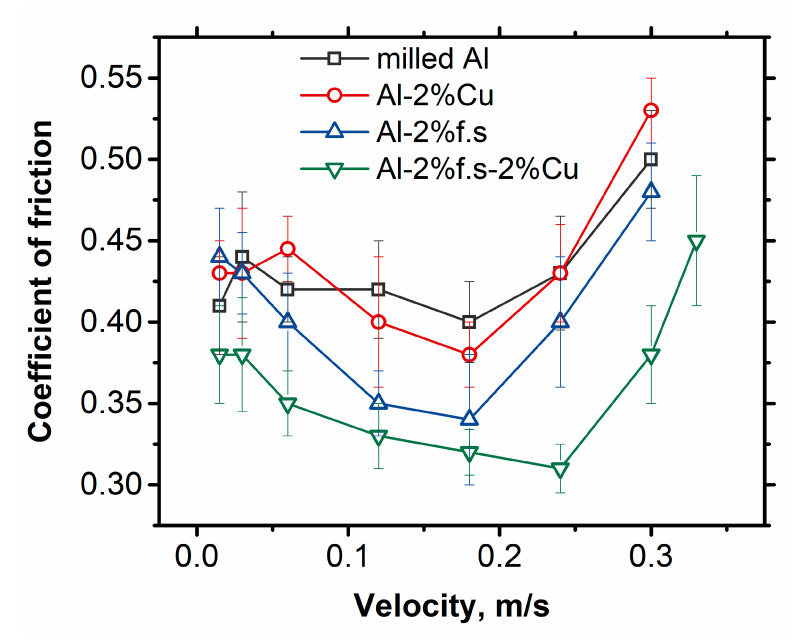
The dependence of the friction coefficient on rotation speed at a load of 20 N (0.13 MPa).

Figure 5 shows plots of the friction coefficient of the materials versus sliding speed.

For pure Al, Al-2%Cu, and Al-2%f.s at a sliding speed of 0.33 m/s, a micro-seizure was observed, so the data are only for Al-2%Cu-2%f.s.

**Figure 6:**
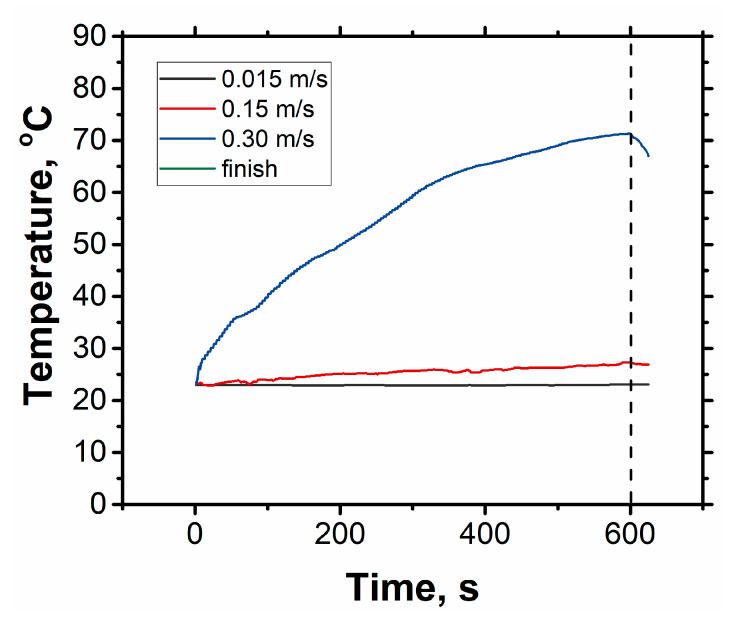
The change in temperature with time at sliding speeds of 0.03, 0.15, and 0.30 m/s for sample Al-2%f.s-2%Cu at a load of 20 N (0.13 MPa).

For all samples, the dependence has the same pattern: with increasing velocity, the friction decreases to a minimum at velocity of about 0.2 m/s and then increases. Three stages can be distinguished, which are all fundamentally different in terms of the friction conditions [20]. In the first stage, at friction velocities up to 0.05 m/s, the contact time between the rubbing surfaces is sufficient to form a complete frictional contact due to the development of plastic microdeformations in the contact zones. In the next stage, from 0.05 to 0.2 m/s, the contact time decreases so much that there is no complete frictional contact formed and the friction decreases. The heat generated by the friction dissipates between the rubbing bodies and into the surrounding space. In the third region, at rotation speeds of 0.2 m/s and above, the frictional contact between the bodies continues to remain unformed, but the heat released in the friction zone has no time to dissipate into the environment and leads to the overheating of the material. The heated surface undergoes plastic deformation to a higher extent, resulting in an increase in the friction force. As shown in Figure 6, friction at a high velocity leads to the considerable heating of the surface.

The addition of fullerene soot results in a reduction in the coefficient of friction and the maximum effect is observed for the 0.12–0.18 m/s speed interval. At 0.12 m/s, the friction coefficient decreases by about 20% compared to that of pure aluminum. The Al-2%f.s-2%Cu composite has lower values at all three stages and the maximum effect is observed for the third stage from a 0.24 m/s rotation speed and higher; meanwhile, the minimal friction coefficient is measured at 0.24 m/s.

**Figure 7:**
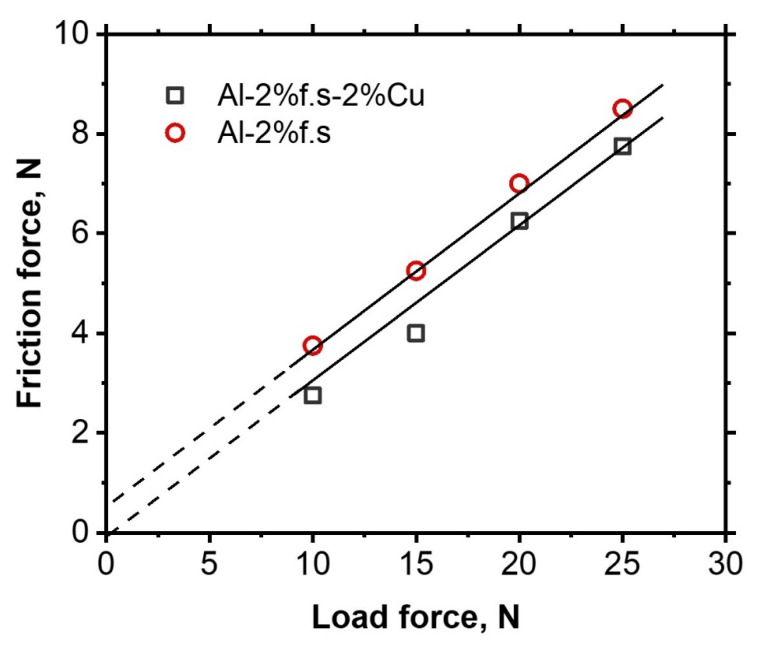
The dependence of friction force on an applied load at a sliding speed of 0.12 m/s for Al-2%f.s and Al-2%f.s-2%Cu.

Figure 7 shows the dependence of the friction force on the applied load for the composites Al-2%f.s and Al-2%f.s-2%Cu.

According to the two-member mathematical model of friction proposed by Deryagin in [21,22], the force of external friction (*F_f_*) is the sum of two components, with the first summand depending on the reaction of the Born forces to the load ($f_{t}\cdot F_{N}$) and the second summand depending on the reaction of repulsive forces to the forces of molecular attraction ($f_{t}\cdot F_{M}$) [22], as seen in the following:

(2) $$F_{f}=f_{D}\cdot \left(F_{N}+F_{M}\right)=f_{D}\cdot F_{N}+f_{D}\cdot F_{M}$$

where *f_D_ =* (*dF_f_*)⁄(*dF_N_*)—differential coefficient of friction; *F_N_*—normal load; *F_M_*—the net force of molecular attraction of the rubbing solids. The dependence of the friction force on the normal load for the composite material Al-2%f.s has the form:

*F_f_* = 0.32(*F_N_* + 1.64) (3)

where it follows that the net force of molecular attraction was 1.64 N and *f_D_* = 0.32. The dependence of the friction force on the normal load for the composite Al-2%f.s-2%Cu looks like:

*F_f_* = 0.3(*F_N_* + 0) (4)

where it follows that the net force of molecular attraction was 0 and *f_D_* = 0.3. The addition of copper promoted the neutralization of the molecular interaction of the friction surfaces. Thus, the addition of copper helps to improve the frictional interaction conditions.

**Figure 8:**
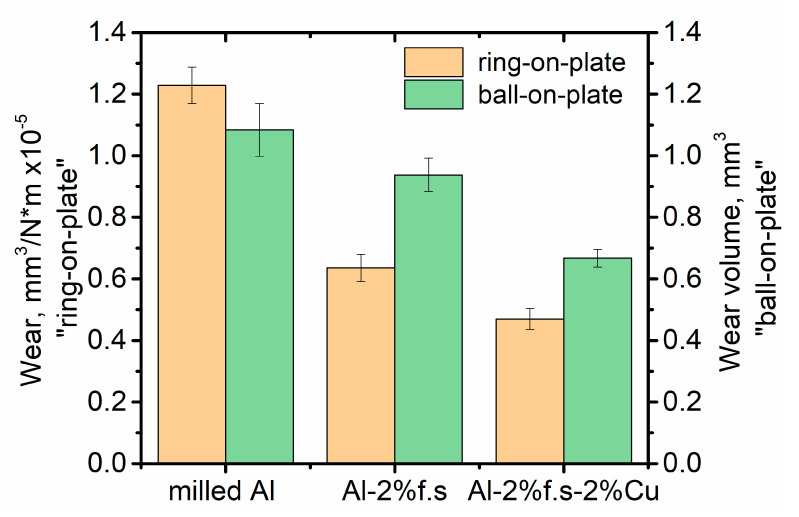
Wear of the samples, measured for the “ring-on-plate” and “ball-on-plate” schemes. Wear volume for “ball-on-plate” scheme was measured for 15 min of the experiment.

The wear resistance of the composites was tested in two ways. Figure 8 shows both the data on the mass loss (ring-plane test scheme) and on the diameter of the wear spot (ball-on-plate test scheme). Both methods demonstrated similar results. The composite Al-2%f.s-2%Cu has the highest wear resistance, followed by Al-2%f.s.

**Figure 9:**
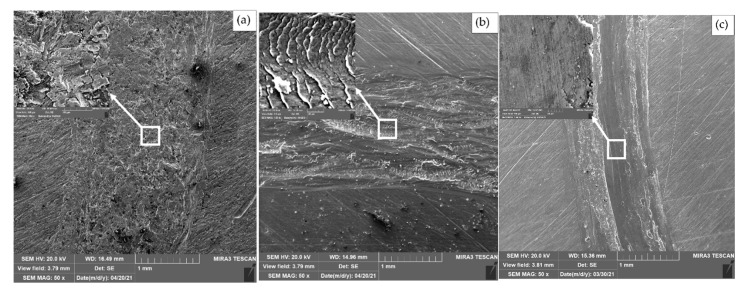
SEM images of the wear track of Al milled (**a**), Al-2%f.s (**b**), and 2%f.s-2%Cu (**c**) samples.

Figure 9 shows scanning electron micrograph images of the typical worn surfaces of the Al milled, Al-2%f.s, and Al-2%f.s-2%Cu samples. 

In the case of the Al milled samples, the predominant wear mechanism is adhesive wear. After the addition of fullerene soot to the aluminum matrix, the wear mechanism changed to an oxidative one with micro-cutting [23,24]. The partial destruction of the oxide film is observed in this specimen, and as a result delamination is visible in these areas. Closer examination shows that the wear marks are relatively smooth and oriented perpendicular to the sliding direction compared to what was also observed in [25]. After the addition of copper to Al-2%f.s, the wear traces became smooth, there was no considerable delamination observed. The wear mechanism in this case was also oxidative, but unlike Al-2%f.s, in this sample, the destruction of the oxide film occurred to a lesser extent. It is possible that the rate of destruction of the oxide film is less than the rate of its recovery.

**Figure 10:**
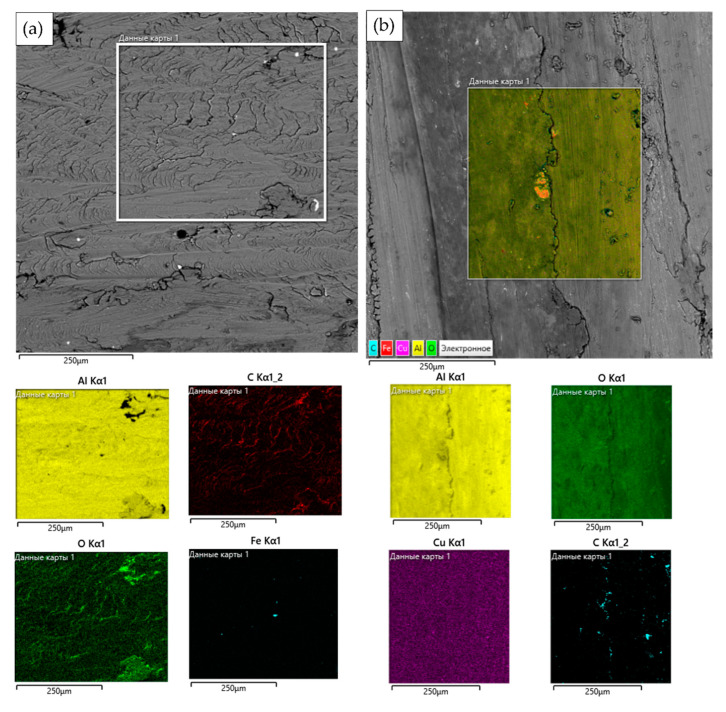
EDS analysis of the worn surfaces of the composites Al-2%f.s (**a**) and Al-2%f.s-2%Cu (**b**).

Figure 10 shows the results of the energy dispersive analysis of the worn surfaces. A significant amount of oxygen was observed on the worn surface of the composites. However, on the worn surface of Al-2%f.s-2%Cu, oxygen is evenly distributed, in contrast to Al-2%f.s, where oxygen does not cover all the surface but is present only in local segregations. This observation confirms the assumption that the oxide film of Al-2%f.s-2%Cu is not destroyed under this friction condition.

A significant amount of oxygen was observed on the worn surface of the composites. However, on the worn surface of Al-2%f.s-2%Cu, oxygen was evenly distributed, in contrast to Al-2%f.s, where oxygen did not cover all of the surface but is present only in local segregations. This observation confirms the assumption that the oxide film of Al-2%f.s-2%Cu was not destroyed under this friction condition.

The debris of the test was black in color. The predominant black color confirms that the aluminum particles were covered with a carbon film, which also stained the white paper. As suggested in [26], it is concluded that the carbon film formed during friction acted as a solid lubricant and had a great influence on the wear characteristics achieved.

**Figure 11:**
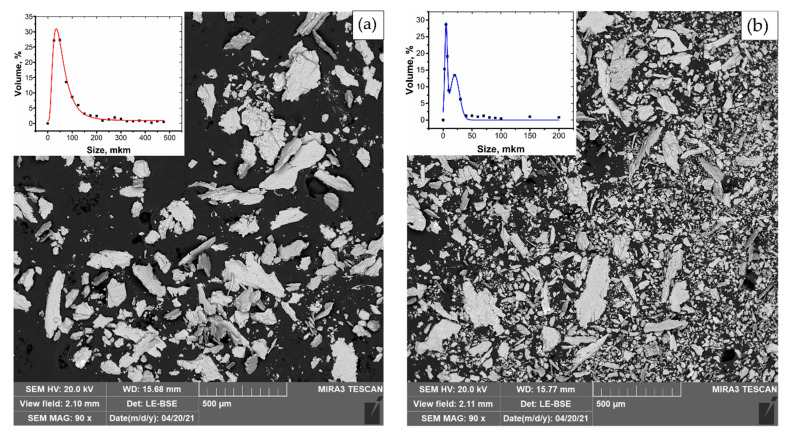
SEM analysis of debris of Al-2%f.s composites (**a**) and Al-2%f.s-2%Cu (**b**).

Figure 11 shows micrographs of the debris of the composites.

The debris of Al-2%f.s composite consists mainly of large detached flakes with an average size of about 60 microns; the fine fraction is practically absent. For the Al-2%f.s-2%Cu composite, fine flakes with an average size of 5 μm are predominant. This explains the lower wear rate of this material. In [27], it is noted that debris of a size less than 5 microns indicates low friction and wear.

## 4. Conclusions

Aluminum-based composite materials reinforced with fullerene soot and with additional alloying with copper were developed. The addition of fullerene soot has been shown to reduce the coefficient of friction by about 20% at a 0.12 m/s rotation speed. Additional alloying with copper reduces the friction coefficient by about 30% at a 0.12 m/s rotation speed compared to that of pure aluminum and leads to a decrease in friction fluctuation. The wear rate of the Al-2%f.s composite decreased twofold compared to that of pure aluminum. With additional alloying with Cu, it reduced 2.5 times.

The analysis of friction vs. load dependence, using the model of friction proposed in [21,22], showed that the Al-2%f.s composites had an adhesion component equal to 1.64 N. With the addition of copper, this component decreased to zero, while the load-dependent differential friction coefficient remained constant.

The wear mechanism of Al-2%f.s and Al-2%f.s-2%Cu composites with respect to that of pure aluminum changed from adhesive to oxidative with micro-cutting. The oxide film of Al-2%f.s-2%Cu was not completely destroyed during friction, leading to less delamination and a higher wear resistance compared to Al-2%f.s. The debris size of the Al-2%f.s-2%Cu composites was much smaller than that of the Al-2%f.s composite, which indicates its low friction and wear.

## Figures and Tables

**Table 1:** Density and hardness of the compact composites.

Sample	Theoretical Density g/cm ^3^	Hydrostatic Density g/cm ^3^	Relative Density %	Hardness kgf/mm^2^
Al-initial	2.7	2.68	99.39	35
Al-milled	2.7	2.66	98.51	50
Al-2%Cu	2748	2713	98.92	135
Al-2%f.s	2683	2635	98.60	165
Al-2%f.s-2%Cu	2720	2693	99.25	240

## Data Availability

Not applicable.
